# Effects of Self-Regulation vs. External Regulation on the Factors and Symptoms of Academic Stress in Undergraduate Students

**DOI:** 10.3389/fpsyg.2020.01773

**Published:** 2020-08-26

**Authors:** Jesús de la Fuente, Francisco Javier Peralta-Sánchez, Jose Manuel Martínez-Vicente, Paul Sander, Angélica Garzón-Umerenkova, Lucía Zapata

**Affiliations:** ^1^School of Education and Psychology, University of Navarra, Pamplona, Spain; ^2^School of Psychology, University of Almería, Almería, Spain; ^3^School of Psychology, Teesside University, Middlesbrough, United Kingdom; ^4^School of Psychology, Fundación Universitaria Konrad Lorenz, Bogotá, Colombia; ^5^Educational Psychologist, Cardiff Council, Cardiff, United Kingdom

**Keywords:** SRL vs. ERL theory, stress factors, stress symptoms, university, academic stress

## Abstract

The SRL vs. ERL theory has shown that the combination of levels of student self-regulation and regulation from the teaching context produces linear effects on achievement emotions and coping strategies. However, a similar effect on stress factors and symptoms of university students has not yet been demonstrated. The aim of this study was to test this prediction. It was hypothesized that the level of student self-regulation (low/medium/high), in interaction with the level of external regulation from teaching (low/medium/high), would also produce a linear effect on stress factors and symptoms of university students. A total of 527 undergraduate students completed validated questionnaires about self-regulation, regulatory teaching, stress factors, and symptoms. Using an *ex post* facto design by selection, ANOVAs and MANOVAs (3 × 3; 5 × 1; 5 × 2) were carried out. The results confirmed that the level of self-regulation and the level of regulatory teaching jointly determined the level of stress factors and symptoms of university students. Once again, a five-level heuristic of possible combinations was configured to jointly determine university students’ level of academic stress. We concluded that the combination of different levels of student regulation and regulation from the teaching process jointly determines university students’ level of academic stress. The implications for university students’ emotional health, stress prevention, and well-being are established.

## Introduction

In university students, stress can be the cumulative emotional result of academic work, future uncertainty, difficulties forming interpersonal relationships, self-doubt, and so on (Chao, 2012). Adjustment to the rigors of university life can be difficult due to the social strain of attending college, along with the student’s renewed independence; in fact, university-related stress has been identified as normative among the general population of college students (Brougham et al., 2009). While stress is “normative” during this developmental period, it is often found to persist afterward, given that the period of university studies is a sensitive moment in one’s lifetime. This type of stress has been analyzed from a clinical health perspective. Stress experienced at university increases one’s susceptibility to mental health problems like depression (Cavazos et al., 2010; Bolin et al., 2017), which can be equally detrimental to one’s satisfaction with school and with life (Jenkins et al., 2013).

### Academic Stress: Definition, Symptoms, and Factors

#### Definition of Academic Stress

Academic stress, as a factor detrimental to psychological health or emotional well-being, is a highly current research topic in the university sphere (Gross, 2008, 2014, 2015a,b; Freire et al., 2018). Many recent studies reveal that academic stress must be kept to an adequate level that allows the university experience to be rewarding for students (Freire et al., 2018). Excessive, repeated stress experiences may place a strain on the student’s emotional well-being during the teaching–learning process (D’Mello, 2013; Shannon et al., 2019). However, this reality has been primarily analyzed from the perspective of clinical and health psychology (Murphy et al., 2005; Reyes-Rodríguez et al., 2013; Lardier et al., 2020; Páramo et al., 2020), and less so from the standpoint of educational psychology. Even university training and intervention programs have traditionally been focused on improving stress management from the student’s perspective (California Polytechnic State University Academic Skills Center, 2020). This report aims to provide a new theoretical approach, as well as associated empirical evidence, to analyze the reality of university stress within the framework of how university teaching and learning processes are carried out.

Academic stress in particular is considered to be the process whereby students view themselves as overwhelmed by academic tasks, hard-pressed to meet academic demands and the requirements for adequate achievement (Frenzel et al., 2016, 2018; Karaman et al., 2017). Some researchers have already highlighted a relationship between general stress and academic stress in activities such as test taking, homework, and class participation (Goetz et al., 2014; Gentsch et al., 2018; Pozos-Radillo et al., 2014).

#### Symptoms of Academic Stress

Previous literature has clearly enumerated the physical and psychological dimensions of stress experiences (Schat et al., 2005). There is plentiful evidence that links stress to negative health conditions (Shaw et al., 2017). Also reported recently is the role of rumination and negative affect, after stressful experiences, in the process of finding meaning (Kamijo and Yukawa, 2018). However, stress as a *response* refers to the physiological, emotional, or behavioral manifestations caused by stressors (Selye, 1978). Similarly, when examining the effects of stress, evidence has shown how stress relates to emotional, behavioral, and cognitive symptoms (Scharp and Dorrance, 2017; Berry, 2020; Di Benedetto et al., 2020).

The *academic stress response* refers to the physiological, emotional, or behavioral manifestations prompted by stressors (Fimian et al., 1989). An acute stressor can trigger various physiological responses (rapid cardiovascular activation, raised blood pressure, increased respiratory rate and corticosteroid levels, sweating, tremor, headaches, weight loss or gain, body aches, and sleep quality). It also prompts a subjective experience related to cognitive reactions (perceived stress, negative thoughts, worry, and feeling of uncontrollability) and negative affect (irritability, agitation, fear, anxiety, and guilt) and can generate behavioral responses [crying, abuse of self and others, smoking (Garett et al., 2017)].

#### Factors of Academic Stress in the Teaching–Learning Process

From the perspective of *educational psychology*, it seems reasonable to assume that academic stress factors at university may originate either in the student or in the context. Stress can be conceptualized in various ways.

It is well known that *stress factors* in the sphere of education are multidimensional, whereas research has tended to address *student-centered factors*, such as personality (Saklofske et al., 2012), ways of coping (Chartier et al., 2011; Freire et al., 2018), student anxiety (Putwain, 2018; Cassady et al., 2019), and student goals (Cabanach et al., 2008; Rusk et al., 2011). Recent research has established consistent *student factors* in this process, such as self-beliefs (Lazarus, 1999), temperament (Hirvonena et al., 2019), test anxiety level (Putwain and Pescod, 2018), and self-regulation behavior (Boyraz et al., 2016; de la Fuente et al., 2020). Stress factors in the *learning process* have also been considered, such as presentations in class, overload of assigned work, team-based assignments, and testing situations (Cabanach et al., 2008; Pozos-Radillo et al., 2014).

The analysis of *context-centered factors*, however, has been more limited, despite certain partial attempts to approach this phenomenon. Stress as a *stimulus* refers to the event or circumstance that has the capacity to trigger emotional reactions in the subject. This is usually external to the subject and can alter the physiological and psychological balance. In reference to the teaching process in particular, factors such as the teacher’s behavior or well-organized teaching have appeared as predictors of emotional well-being and student engagement, reducing the level of stress (Frenzel et al., 2018; Lekwa et al., 2018; Krijgsman et al., 2019; Shannon et al., 2019).

Among academic stressors, three main groups can be distinguished: (1) those related to evaluation processes, (2) those related to work overload, and (3) other conditions of the teaching–learning process, such as social relationships (teacher–student and peer relationships), teaching methodology, and various organizational components (inadequate study plans, scheduling problems, overlapping programs, low student participation in organization and decision making, overcrowding, etc.) (González-Cabanach et al., 2016, 2017, 2018). Denovan and Macaskill (2013), in a study that lists 11 potential situations that generate stress and stress symptoms, found that the situations predictive of chronic stress were class participation, required assignments, and test taking. Bob et al. (2014), in a sample of medical students, found that the top stressors were exams, falling behind in the learning schedule, the large amount of content to be learned, heavy workload, and lack of time to review what has been covered.

### SRL vs. ERL Theory as a Heuristic for Analyzing Stress in the Teaching–Learning Process

Stress factors and effects can also be conceptualized from an *interactive approach*, which speaks of the joint, combined effect of student factors and of factors pertaining to the teaching process that the student is exposed to. Previous research has reported effects from the learning context, referring to factors such as regulation carried out through the teaching process (Vermunt, 1989, 2007). This is the approach taken in the present study. This view is important because it allows academic stress to be addressed from two directions, from the subject and the context, in combination. The theory of *self-regulated learning vs. externally regulated learning* (de la Fuente, 2017) can serve as a research heuristic for analyzing this interaction. It is based on certain assumptions:

(1) University students can have prior *personal* characteristics that make them less susceptible to suffering stress experiences. *Self-regulation* behavior, as a meta-behavioral variable (de la Fuente, 2015; Craig et al., 2020), can be considered a *personal protective factor* against stress. Previous evidence has shown that behavioral self-regulation is positively associated with greater resilience (Artuch-Garde et al., 2017), a higher level of positive emotionality, and less negative emotionality (de la Fuente et al., 2017), as well as greater use of problem-focused strategies and less use of emotion-focused strategies (de la Fuente et al., 2015c). Also, Self-regulation is negatively associated with the surface learning approach, negative emotionality, and emotion-focused strategies for coping with stress (de la Fuente et al., 2020). The presence of self-regulation behavior can be classed as *high* (good *self-regulatory behavior*), leading to a lower perception of stress factors and symptoms; *middle* (*non-regulatory behavior*), leading to their perception at a medium level; and *low* (*dysregulatory behavior*), which leads to a high perception of stress factors and symptoms. Consequently, a high level of self-regulation operates as a protective factor and a low level of self-regulation as a risk factor for stress.
(2) Similarly, an adequate teaching process (*effective teaching*) can be considered a *contextual protective factor* against stress because it favors the student’s perception of control over the learning process (Azevedo et al., 2008; Goe et al., 2008; Roehrig et al., 2012). Previous evidence has shown that *high regulatory teaching* (effective teaching) is a *protective factor* against stress, because it is positively associated with a higher level of positive emotionality and lower negative emotionality (de la Fuente et al., 2017), as well as with greater use of problem-focused strategies and less use of emotion-focused strategies (de la Fuente et al., 2017). Similarly, *low regulatory teaching* is a risk factor for stress because is positively associated with surface learning, negative emotionality, and emotion-focused coping with stress (de la Fuente et al., 2020). The *low/medium/high level of external regulation from the teaching context* will function as a contextual protective or risk factor for stress. If the teaching includes a high level of external regulation (*good external regulation*), it will predispose to low stress, since the teaching–learning process is designed and developed in a way that offers protection from stress. By contrast, if external regulation is absent (*external non-regulation*), this mid-level option will allow a medium level of stress factors and symptoms to appear, originating from the teaching and learning process. Finally, if the teaching produces *external dysregulation*, this lowest level of external regulation would predispose to the appearance of a high level of stress factors and symptoms.
(3) It is therefore possible to analyze the combination of the two preceding factors (personal × contextual) in order to determine the probable level of protection or risk for stress that results. The *combination of personal and contextual* factors, whether they are *protective or risk factors*, can help determine university students’ perceived level of stress factors and symptoms. Thus, for example, the combination of low student self-regulation with low regulation from teaching (risk factors in both cases) would predispose to a high level of stress factors and symptoms in students. However, high student self-regulation combined with high regulation from teaching, both protection factors, would predispose to a low level of stress factors and symptoms. The possible combinations have been established in a *five-level heuristic* that calculates the regulation level that exists in the student–teacher interaction (de la Fuente et al., 2019b, p. 12; de la Fuente et al., 2020, p. 5).

The five-level heuristic was created through a process of several steps. First, students’ low/medium/high levels of self-regulation were determined. Second, low/medium/high levels of regulatory teaching were established. Third, a combined regulation level was calculated by averaging these two regulation levels (each with values of 1–3); these averages were then assigned ranks from 1 to 5 (for the averages 1.0, 1.5, 2.0, 2.5, 3.0). Fourth, each rank was given a descriptive name according to its combination values, ranging from high dysregulation to high regulation. Fifth, each rank was also labeled with its corresponding value as a risk or protection factor against stress. See Table 1.

**TABLE 1:** Combinations between the model parameters hypothesized by SRL vs. ERL theory (de la Fuente et al., 2019b, 2020, p. 5).

Combination level		Regulation		Regulation tendency	Stress protection	Stress risk
SR level (range)	RT level (range)	Average/rank				
**3** (3.85–5.00) **H**	**3** (2.84–5.00) **H**	3.0	**5**	**High–High:** *High-regulation*	*High protector*	*Low risk*
**2** (3.10–3.84) **M**	**3** (2.84–5.00) **H**	2.5	**4**	**Medium–High**: *Regulation*	*M-H protector*	*M-L risk*
**3** (3.85–5.00) **H**	**2** (2.35–2.83) **M**	2.5	**4**	**High–Medium**: *Regulation*	*M-H protector*	*M-L risk*
**2** (3.10–3.84) **M**	**2** (2.35–2.83) **M**	2.0	**3**	**Medium:** *Non-regulation*	*Medium protector*	*M risk*
**2** (3.10–3.84) **M**	**1** (1.00–2.34) **L**	1.5	**2**	**Medium–Low**: *Dysregulation*	*M-L protector*	*M-H risk*
**1** (1.00–3.09) **L**	**2** (2.35–2.83) **M**	1.5	**2**	**Low–Medium**: *Dysregulation*	*M-L protector*	*M-H risk*
**1** (1.00–3.09) **L**	**1** (1.00–2.34) **L**	1.0	**1**	**Low–Low:** *High Dysregulation*	*Low protector*	*High risk*

*L, low; M, medium; H, high; SR, self-regulation; RT, regulatory teaching. 1–5 (Rank of regulation).*

### Aims and Hypothesis

Based on our previous research and findings, our research team sought to validate the combination of different types of regulation, assuming that no linear models previously found would be applicable to the factors and symptoms of stress. This line of research has provided prior empirical evidence that the *five heuristic levels* derived from SRL vs. ERL theory have the potential to explain other differences. For example, these levels have been used to explain university students’ experience of positive versus negative achievement emotions (de la Fuente et al., 2017); the type of stress-coping strategies they use, whether emotion- or problem-focused (de la Fuente et al., 2020); their learning approaches; and even academic achievement^1^. It remains to be seen, therefore, whether this heuristic can be shown to determine levels of stress factors inherent in the teaching–learning process and the stress symptoms produced. This is the aim of the present study.

Consequently, our *specific objectives* were as follows: (1) to establish whether the regulation levels of the student and of the teaching process determined *academic stress factors and symptoms of stress* and (2) to determine whether the interaction of these levels, as described in SRL vs. ERL theory, were associated with levels of stress factors and symptoms. The corresponding *hypotheses* were established: (1) low/medium/high levels of regulation in students and in their teaching process will result in a corresponding low/medium/high level of academic stress factors and symptoms; (2) the lower the combination rank of student and teaching regulation, the higher the factors and symptoms of academic stress because of the greater presence of risk factors, and vice versa.

## Materials and Methods

### Participants

The participants were 527 undergraduate students from two Spanish public universities. The sample was composed of students enrolled in psychology and primary education degree programs; 82.6% were women, and 17.4% were men. Their ages ranged from 19 to 25, with a mean of 22.15 ([σ_*X*_] = 7.1) years. Sampling was incidental and not probabilistic, since the sample could not be randomized. The students came from nine class subjects (specific teaching–learning processes), whose teachers desired to participate and had invited them. As is common in these types of degree programs, the sample contains a large majority of women. In some cases, students did not complete all the inventories, or some instruments were only partially completed. This explains the variability in the number of participants in the different analyses.

### Instruments

#### Learning Process

##### Self-regulation Behavior

This variable was measured using the *Short Self-Regulation Questionnaire* (*SSRQ*) (Miller and Brown, 1991). It has already been validated in Spanish samples (Pichardo et al., 2014, 2018) and possesses acceptable validity and reliability values, similar to the English version (Garzón-Umerenkova et al., 2017). The Short SRQ is composed of four factors (goal setting–planning, perseverance, decision making, and learning from mistakes) and 17 items. All items have saturations greater than 0.40, with a consistent confirmatory factor structure [chi-square or CMIN = 250.83, df = 112, *p* < 0.001; relative chi-square, CMIN/df = 2,239; SRMR = 0.0420; comparative fit index (CFI) = 0.90, TLI = 0.92, normed fit index (NFI) = 0.90, root mean square error of approximation (RMSEA) = 0.05]. Internal consistency was acceptable for the total of questionnaire items (α = 0.86) and for the factors of goal setting–planning (α = 0.79), decision making (α = 0.72), learning from mistakes (α = 0.72), and perseverance (α = 0.73).

#### Teaching Process

##### Regulatory teaching

*The Scales for Assessment of the Teaching–Learning Process*, *ATLP*, *student version* (de la Fuente et al., 2012), was used to evaluate students’ perception of the teaching process. The scale entitled *Regulatory Teaching* is Dimension 1. IATLP-D1 comprises 29 items structured along five factors: specific regulatory teaching, regulatory assessment, preparation for learning, satisfaction with the teaching, and general regulatory teaching. The ATLP is a self-report instrument to be completed by the teacher and the students, available in Spanish and English versions. It also includes a qualitative part where students can make recommendations for improving each of the processes evaluated. As for the instrument’s external validity, results are consistent; there are different interdependent relationships between perceptions of variables found in the academic environment. The scale was validated in university students (de la Fuente et al., 2012) and showed a factor structure with adequate fit indices (chi-square or CMIN = 490.626, *df* = 98, *p* < 0.001; relative chi-square or CMIN/df = 5,00; SRMR = 0.0802, CFI = 0.958, TLI = 0.959, NFI = 0.950, NNFI = 0.967, RMSEA = 0.068) and adequate internal consistency (IATLP-D1: α = 0.83; specific regulatory teaching, α = 0.897; regulatory assessment, α = 0.883; preparation for learning, α = 0.849; satisfaction with the teaching, α = 0.883; and general regulatory teaching, α = 0.883).

##### Factors of stress

*Academic Stress Questionnaire*, *CEA* (Cabanach et al., 2008). We analyzed the internal structure of the scale. In order to verify the second-level structure, a confirmatory factor analysis (CFA) was conducted on the whole set of data from our sample. The default model shows good fit [chi-square or CMIN = 66,457, df = 13, *p* < 0.001; relative chi-square or CMIN/df = 5,11; SRMR = 0.075, CFI = 0.935, TLI = 0.961, IFI = 0.947, RFI = 0.965, NFI = 0.947, RMSEA = 0.057, HOELTER = 0.430 (*p* < 0.05) and 0.532 (*p.* < 01)]. The model proposed for this version of the scale contains 53 items with a structure of seven factors and two dimensions, with one factor different from the original version. The resulting factors, in two dimensions, were: (1) *Dimension of Stress in Learning:* task overload (Factor 2), dif. perform. control (F3), social climate (Factor 5), and test anxiety (Factor 7) and (2) *Dimension of Stress in Teaching*: method. difficulties (Factor 1), Public speaking (Factor 4), content lacks value (Factor 6). Overall reliability = 0.961; part 1 = 0.932, part 2 = 0.946.

#### Learning Product

##### Symptoms of academic stress

*Stress Response Questionnaire*, *CRE* (Cabanach et al., 2008). The psychometric properties of this scale were adequate in this sample of Spanish students. The factors of the Confirmatory Structural Model of the CRE were: chi-square or CMIN = 846.503, DF (375-76) = 299, *p* < 0.001; relative chi-square, CMIN/df = 2,831; SRMR = 0.0721, NFI = 0.952, RFI = 0.965, IFI = 0.953, TLI = 0.951; F1, *burnout*; F2, *sleep difficulties*; F3, *irritability;* F4, *negative thoughts*; and F5, *agitation.* The unidimensionality of the scale and metric invariance in the assessment samples were confirmed [RMSEA = 0.046; CFI = 0.922 and TLI = 0.901; HOELTER = 431 (*p* < *0.05*) and 459 (*p* < 0.01)]. Cronbach’s alpha was 0.920; part 1 = 0.874, and part 2 = 0.863.

### Procedure

The University Guidance department at the two universities invited teachers of different subjects to participate in the research. Once the teachers accepted, they were given full information about the research project. They in turn invited their students to participate by completing the scales. Participants voluntarily completed the scales using an online platform^2^ (de la Fuente et al., 2015a). As part of their initial registration on the platform, students read and signed their informed consent. The platform then assigned a randomly generated participant code to each student, so anonymity was maintained. Students received a Certificate of Participation in the research project for completing the inventories outside of regular class hours; participation time was shown on the certificate (a total of 2 h). These certificates were unrelated to the ECTS credits for the subject. Students were required to complete all the questionnaires in order to receive the certification.

The assessments covered a total of five specific teaching–learning processes of different university subjects that occurred over two academic years. *Self-regulation behavior* was evaluated in September–October 2018 and 2019, *regulatory teaching* process variables in February–March 2018 and 2019, and *factors and symptoms of stress* in May–June 2018 and 2019. The procedure was approved by the respective Ethics Committees of the two universities, in the context of an R&D project (2018–2021) and UAL18 SEJ-DO31-A-FEDER (2018–2021).

### Data Analysis

#### Research Design

An *ex post* facto, non-linear, inferential-type design was used. This design has provided evidence that aligns with SRL vs. ERL theory. A linear prediction is not intended; instead, we attempt to demonstrate inferential, interdependence relationships between levels of the different variables. The levels refer to H/M/L in self-regulation × H/M/L in regulatory teaching (3 × 3), and the combination level 1/2/3/4/5 according to the heuristic (5 × 1). This design seemed best suited to demonstrating the effect of each of the combinations hypothesized in the five-level heuristic.

#### Preliminary Analysis

A preliminary *CFA* was performed on this sample as evidence of factor validity and to ensure the prior structural adjustment of each inventory using the AMOS statistical program (v. 22). Reliability (Cronbach’s alpha) was also estimated using SPSS (v. 25). The following were used for analysis of the CFA model:

(1) Discrepancy functions, such as the chi-square test (or CMIN in the AMOS program), relative chi-square (CMIN/df less than 5; Schumacker and Lomax, 2004). SRMR should be less than 0.08 (Browne and Cudeck, 1993), and ideally less than 0.05. Alternatively, the SRMR’s upper confidence interval should not exceed 0.08 (Hu and Bentler, 1995).
(2) Tests that compare the target model with the null model, such as the CFI, NFI, TFI, and IFI. The NFI should exceed 0.90 (Byrne, 1994) or 0.95 (Schumacker and Lomax, 2004), the goodness of fit index (GFI) should exceed 0.90 (Byrne, 1994), and the CFI should exceed 0.93 (Byrne, 1994). In general, index values equal to or greater than 0.90 and 0.95, respectively, were taken to indicate acceptable and close fit to the data (McDonald and Marsh, 1990). In addition, the RMSEA was used. RMSEA values equal to or less than 0.08 and 0.05 were also taken to indicate acceptable and close levels of fit (Jöreskog and Sörbom, 1993).

#### Typology of Five Combinations According to the Heuristic

The procedure for forming the low/medium/high groups has already been presented in previous work (de la Fuente et al., 2019b, 2020). Basically, it consisted of a cluster analysis followed by simple and multiple ANOVAs to delimit the significant differences between the different levels of regulation. The exact cutoff points are shown in Table 2.

**TABLE 2:** Combinations between the model parameters hypothesized by SRL vs. ERL theory, for Factors and Symptoms of Academic Stress (de la Fuente et al., 2019b, 2020, p. 5).

Regulation levels		Regulation		Regulation tendency	Factors of stress*	Symptoms of stress*
SR level (range)	RT level (range)	Average/rank				
**3** (3.85–5.00) **H**	**3** (2.84–5.00) **H**	3.0	**5**	**High–High:** *High-regulation*	*Low*	*Low*
**2** (3.10–3.84) **M**	**3** (2.84–5.00) **H**	2.5	**4**	**Medium–High**: *Regulation*	*M-L*	*M-L*
**3** (3.85–5.00) **H**	**2** (2.35–2.83) **M**	2.5	**4**	**High–Medium**: *Regulation*	*M-L*	*M-L*
**2** (3.10–3.84) **M**	**2** (2.35–2.83) **M**	2.0	**3**	**Medium:** *Non-regulation*	*M*	*M*
**2** (3.10–3.84) **M**	**1** (1.00–2.34) **L**	1.5	**2**	**Medium–Low**: *Dysregulation*	*M-H*	*M-H*
**1** (1.00–3.09) **L**	**2** (2.35–2.83) **M**	1.5	**2**	**Low–Medium**: *Dysregulation*	*M-H*	*M-H*
**1** (1.00–3.09) **L**	**1** (1.00–2.34) **L**	1.0	**1**	**Low–Low:** *High Dysregulation*	*High*	*High*

H, high; M, medium; L, low. *Effects analyzed in this investigation. 1–5 (Rank of regulation).

The multivariate analyses (MANOVAs) showed a statistically significant main effect of the five interaction types on low/medium/high levels of self-regulation (SR) and of regulatory teaching (RT) (see: de la Fuente et al., 2020, p. 5, and Table 1):

*Combination 1* presented a statistically significant low level in *SR* and low level in *RT* (*1 and 1*). The average regulation level is 1.0, and the rank is 1. The effects are a *high level of stress factors and symptoms*.
*Combination 2* had a statistically significant low level in *SR* and medium level in *RT*, or vice versa (*1 and 2*, *or 2 and 1*). The average regulation level is 1.5, and the rank is 2. The effects are a *medium-high level of stress factors and symptoms*.
*Combination 3* presented a statistically significant medium SR level (*2*) and medium RT level (*2 and 2*). The average regulation level is 2.0, and the rank is 3. The effects are a *medium level of stress factors and symptoms*.
*Combination 4* had statistically significant medium SR and high RT, or vice versa (*2 and 3*, *or 3 and 2*). The average regulation level is 2.5, and the rank is 4. The effects are a *medium-low level of stress factors and symptoms*.
*Combination 5* presented a statistically significant high SR and high RT (*3 and 3*). The average regulation level is 3.0, and the rank is 5. The effects are a *low level of stress factors and symptoms*.

#### Statistical Analyses

First, after checking the sample for adequacy assumptions, simple and multiple multivariate analyses were conducted (ANOVAs and MANOVAs; Pillai’s Trace, partial eta squared, and power) to establish the effect of low/medium/high levels of SR and of RT (IVs) on the factors and symptoms of stress (DVs). To ensure that gender did not have a significant effect, it was initially inserted as an IV in the analyses. As gender did not appear as an independent variable with any significant effect, it was eliminated from the analyses performed. Second, the five-level heuristic was taken as an IV to establish its potential for determining factors and symptoms of stress.

## Results

### Combined Effects of Levels of SR and Levels of RT

#### Effects in Academic Stress Factors

A statistically significant main effect of the *IV SR* H/M/L was noted on the total of *academic stress factors.* No significant statistical effect appeared of the *IV RT* H/M/L on *total academic stress.* No statistical effect of the interaction SR × RT appeared.

Complementarily, a statistically significant main effect of the *IV SR* H/M/L, and an interaction *SR* × *RT* statistically significant effect, was noted in the dimensions of academic stress factors. The statistically significant partial effect was maintained for the *IV SR* H/M/L for both *stress factors of teaching* and *stress factors of learning*. Also, a statistically significant partial effect was maintained for the interaction *SR* × *RT* H/M/L for both *stress factors of learning*. There were no significant interaction effects SR × RT for stress factors in the stress factors of teaching.

A statistically significant partial effect was maintained for the *IV SR* H/M/L for both factors of stress: method difficulties, public intervention, content lacks value, task overload, social climate, difficulties of performance control, and for test anxiety. No significant statistical effect appeared of the *IV RT* H/M/L. Complementarily, a statistically significant partial effect was maintained for the interaction *SR* × *RT* H/M/L for *public intervention*, *content lacks value*, *social climate*, and *test anxiety*. See Table 3.

**TABLE 3:** Combined effects (3 × 3) between the levels of *SR* with levels of *RT* in the *stress factors and symptoms* (*n* = 486).

SR	Low (*n* = 134)			Medium (*n* = 229)			High (*n* = 123)			IV	Effects	
RT	Low	Med	High	Low	Med	High	Low	Med	High		*F*(Pillai’s index)	*post hoc*
				
*n=*	28	76	30	55	110	64	75	45	63			
**Stress factors**												
Total	2.95(0.70)	2.70(0.70)	2.46(0.80)	2.25(0.21)	2.26(0.59)	2.26(0.66)	1.99(0.72)	1.96(0.59)	1.93(0.72)	**SR**	*F*(2,392) = 36.398** η^2^ = 157	
										**RT**	*F*(2,392) = 0.661.^5^**^11^** η^2^ = 0.003	
Teaching process	3.67(0.74)	3.62(0.60)	3.73(0.56)	3.29(0.63)	3.24(0.64)	3.27(0.64)	3.06(0.91)	2.96(0.64)	2.68(0.78)	**SR**	*F*(4,784) = 17.385** η^2^ = 0.081	
Learning process	3.28(0.60)	2.96(0.68)	2.87(0.80)	2.39(0.61)	2.64(0.64)	2.62(0.55)	2.33(0.73)	2.31(0.70)	2.22(0.82)*	**SR × RT**	*F*(8,784) = 2.523** η^2^ = 0.025	
*Factors of teaching process*										**SR**	*F*(2,392) = 29.397*** η^2^ = 0.130	1 > 2 > 3
Method. Difficulties	3.96(0.63)	3.75(0.65)	3.69(0.64)	3.57(0.79)	3.42(0.75)	3.72(0.79)	3.32(1.1)	3.19(0.74)	3.16(0.86)	**SR**	*F*(2,392) = 12.296*** η^2^ = 0.059	1 > 2 > 3
Public interventions	3.31(1.0)	3.92(1.0)	4.05(0.80)	3.38(1.0)	3.39(1.0)	3.30(1.0)	3.30(1.1)	2.84(0.97)	2.68(1.0)*	**SR**	*F*(2,392) = 12.398*** η^2^ = 0.064	1 > 2 > 3
										**SR × RT**	*F*(4,392) = 2.869* η^2^ = 0.028	
Content lacks value	3.74(0.93)	3.18(0.81)	3.46(0.76)	2.91(0.97)	2.92(0.95)	2.78(0.96)	2.54(1.1)	2.84(1.0)	2.19(1.1)	**SR**	*F*(2,392) = 20.779*** η^2^ = 0.096	1 > 2 > 3
										**SR × RT**	*F*(4,392) = 2.932* η^2^ = 0.029	
*Factors of learning process*										**SR**	*F*(2,394) = 27.975** η^2^ = 0.130	1 > 2 > 3
										**SR × RT**	*F*(4,392) = 2.463* η^2^ = 0.025	1 > 2 > 3
Task overload	3.52(0.75)	3.28(0.71)	3.30(1.0)	2.72(0.68)	2.87(0.73)	2.77(0.74)	2.52(0.76)	2.60(0.85)	2.33(0.90)	**SR**	*F*(2,392) = 28.639*** η^2^ = 0.127	1 > 2 > 3
Social climate	2.67(0.98)	2.38(0.90)	2.18(0.83)	1.95(0.80)	2.27(0.82)	2.35(0.82)	2.06(0.74)	2.03(0.81)	2.10(1.0)*	**SR**	*F*(2,392) = 3.347* η^2^ = 0.017	1 > 2 > 3
										**SR × RT**	*F*(4,392) = 2.932* η^2^ = 0.029	
Dif. Perf. control	3.48(0.62)	3.23(0.69)	3.23(0.71)	2.57(0.73)	2.82(0.69)	2.81(0.62)	2.44(0.80)	2.41(0.73)	2.31(0.84)	**SR**	*F*(2,392) = 36.815*** η^2^ = 0.158	1 > 2 > 3
Test anxiety	3.46(0.76)	2.95(0.92)	2.79(0.98)	2.30(0.77)	2.62(0.82)	2.54(0.77)	2.29(1.0)	2.20(0.81)	2.14(0.99)*	**SR**	*F*(2,392) = 22.998*** η^2^ = 0.105	1 > 2 > 3
										**SR × RT**	*F*(4,392) = 3.054** η^2^ = 0.030	
**Stress symptoms**												
Total	2.95(0.70)	2.70(0.70)	2.48(0.80)	2.52(0.71)	2.26(59)	2.26(0.66)	1.99(0.72)	1.96(0.59)	1.93(0.72)	**SR**	*F*(2,477) = 30.609 *** η^2^ = 0.114	1 > 2 > 3
										**SR**	*F*(10,948) = 10.312*** η^2^ = 0.098	1 > 2 > 3
Burnout	3.41(0.79)	3.21(0.81)	3.12(1.0)	2.79(0.93)	2.80(0.89)	2.81(0.89)	2.37(0.71)	2.28(0.82)	2.26(0.87)	**SR**	*F*(2,477) = 27.752*** η^2^ = 0.104	1 > 2 > 3
Sleep difficulties	2.72(0.86)	2.58(0.90)	1.98(1.0)	2.21(0.90)	2.08(0.72)	2.16(0.85)	1.98(1.0)	2.08(0.08)	1.98(0.90)	**SR**	*F*(2,477) = 9.361*** η^2^ = 0.138	1 > 2 > 3
Irritability	2.72(1.0)	2.32(0.92)	2.12(0.86)	2.00(0.87)	2.00(0.72)	1.97(0.73)	1.68(0.64)	1.76(0.73)	1.72(0.82)	**SR**	*F*(2,477) = 17.760*** η^2^ = 0.087	1 > 2 > 3
Negative thoughts	3.31(1.0)	2.94(1.0)	2.75(1.0)	2.18(0.90)	2.33(0.86)	2.14(0.76)	1.96(0.82)	1.82(0.75)	1.83(0.84)	**SR**	*F*(2,477) = 43.362*** η^2^ = 0.164	1 > 2 > 3
Agitation	2.61(0.92)	2.37(0.88)	2.12(0.94)	2.07(0.93)	2.11(0.74)	2.22(0.77)	1.95(0.96)	1.88(0.96)	1.89(0.90)	**SR**	*F*(2,477) = 7.739*** η^2^ = 0.030	1 > 2 > 3

Statistical effect in the interaction of variables: SR, personal self-regulation levels, or RT, regulatory teaching levels. SR, self-regulation effect; RT, regulatory teaching effect. 1 = low level; 2 = medium level; 3 = high level. ***p < 0.001, **p < 0.01, p < 0.05.

#### Effects in Academic Stress Symptoms

A statistically significant main effect of the *SR IV levels* was noted on the total of *academic stress symptoms.* Also, the statistically significant partial effect was maintained for the *IV SR levels* for all *stress symptoms*: burnout, sleep difficulties, irritability, negative thoughts, and agitation. No significant effects appeared for regulatory teaching or for the SR × RT interaction. See Table 3.

### Combination Typology for Understanding Academic Stressors and Stress Symptoms

#### Effects in Academic Stress Factors

**FIGURE 1:**
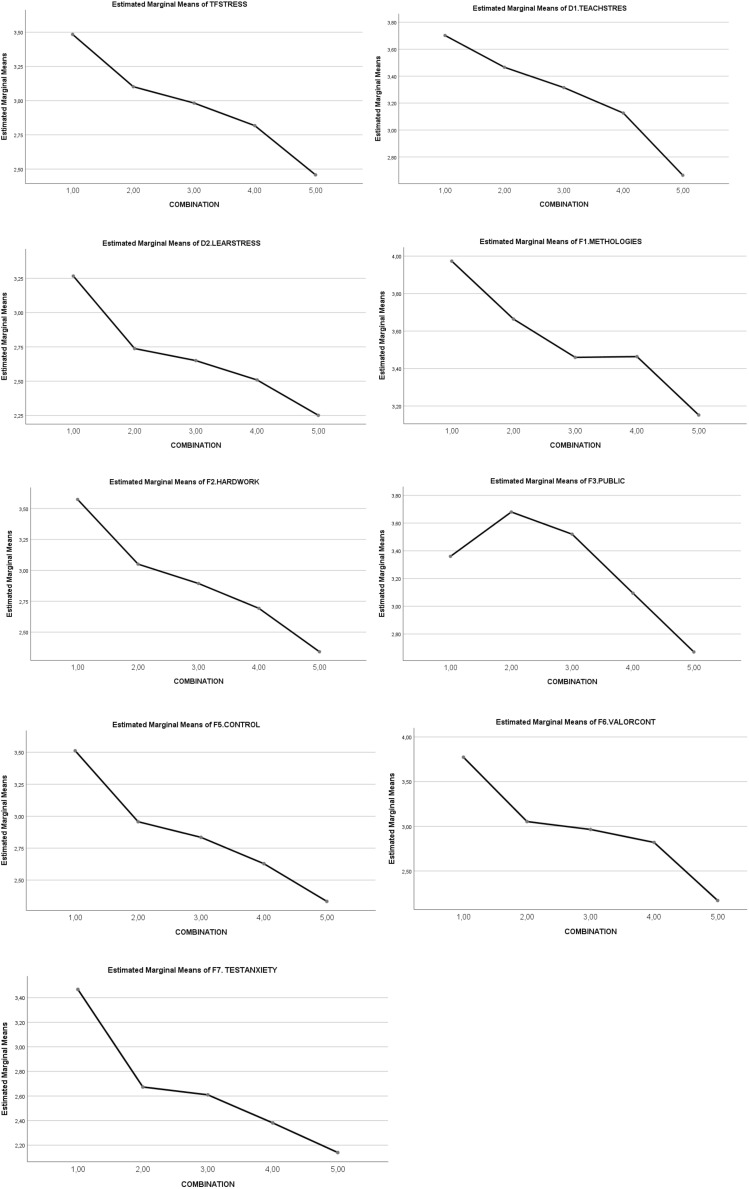
Graphical representation of the effects of the types of combinations (levels 1–5) on *academic stress factors and:* (1) TFSTRESS = total stressors; (2) D1. TEACHSTRES*S* = stressors of teaching process; (3) D2. LEARSTRESS = stressors of learning process; (4) F1. METHODOLOGIES = method. difficulties; (5) F2. HARDWORK = work overload; (6). F3. PUBLIC = public interventions; (7) F5. CONTROL = dif. performance control; (8) F6. VALORCONT = content lacks value; (9) F7. TESTANXIETY = test anxiety.

A statistically significant main effect of the *five combinations of SR and RT* was noted on total *academic stress factors* (5,4 < 3 < 2,1). The statistically significant partial effect was maintained in the *five combinations*, for both teaching factors and learning factors. In the case of *teaching factors*, a significant statistical effect appeared in favor of low levels (5 < 4,3 < 2,1), similarly to the *learning factors* (5,4 < 3,2 < 1). The statistically significant partial effect was maintained for each factor of the teaching process (*method difficulties*, *public interventions*, and *content lacks value*) and for the learning process (work *overload*, *differences in performance control*, and *test anxiety*). See Table 4. The graphic representation of the differential progressive effect of the combination between SR and RT levels is shown in Figure 1. Thus, academic stress factors progressively decrease through the five levels of interaction. Overall, the clearest effect that appears is that a higher interaction level leads to a decreasing level of stress factors.

**TABLE 4:** Effects of types of combination in the factors of stress (*n* = 401).

DVs	Type of Combination in Groups (IVs)					
	1	2	3	4	5	Effects *post hoc*
		
	(*n* = 26)	(*n* = 101)	(*n* = 135)	(*n* = 87)	(*n* = 52)	
**Stress factors**						
Total	3.45(0.52)	3.10(0.53)	2.97(0.61)	2.81(0.57)	2.47(0.75)	*F*(4,396) = 15.207 (Pillay), *p* < 0.001, η^2^ = 0.133;5,4 > 3 > 2,l** *F*(8,792) = 9,124 (Pillay), *p* < 0.001, η^2^ = 0.085
Teaching factors	3.67(0.64)	3.47(0.63)	3.29(0.70)	3.12(0.65)	2.68(0.78)	*F*(4,396) = 15.108, *p* < 0.001, η^2^ = 132; 5 < 4,3 < 2,1**
Learning factors	3.28(0.70)	2.71(0.71)	2.64(0.69)	2.47(0.74)	2.22(0.82)	*F*(4,396) = 11.420, *p* < 0.001, η^2^ = 103; 5,4 < 3,2 < 1** *F*(28,1572) = 3,869 (Pillay), *p* < 0.001, η^2^ = 0.064
Method. difficulties	3.96(0.63)*	3.67(0.71)	3.45(0.80)	3.47(0.81)	3.16(0.86)	*F*(4,396) = 6.089, *p* < 0.001, η^2^ = 0.058, 5 < 2,1**; 4,3 < 1**
Public interventions	3.31(0.98)	3.68(0.99)	3.48(0.99)	3.08(0.97)	2.68(0.96)	*F*(4,396) = 9.425, *p* < 0.001, η^2^ = 0.087, 5,4 < 3,2,1**
Content lacks value	3.74(0.93)*	3.06(0.89)	2.95(0.98)	2.81(0.99)	2.19(0.99)	*F*(4,396) = 12.518, *p* < 0.001, η^2^ = 0.112 5,4 < 3 < 2,l**
Work overload	3.52(0.75)*	3.04(0.78)	2.89(0.80)	2.69(0.79)	2.33(.90)	*F*(4,396) = 12.004, *p* < 0.001, η^2^ = 0.108, 5,4,3 < 2,1**
Social climate	2.67(0.98)	2.19(0.88)	2.23(0.81)	2.20(0.82)	2.10(0.99)	*F*(4,396) = 1.949, *p* < 0.001, η^2^ = 0.019n.s.
Dif. Perf. control	3.48(0.62)*	2.95(0.78)	2.84(0.75)	2.62(0.70)	2.10(0.99)	*F*(4,396) = 12.290, *p* < 0.001, η^2^ = 0.115, 5,4 < 3 < 2,1**
Test anxiety	3.46(0.76)*	2.67(0.91)	2.60(0.88)	2.38(0.80)	2.14(0.99)	*F*(4,396) = 11.191, *p* < 0.001, η^2^ = 0.102, 5,4 < 3,2 < 1**

*p < 0.05; **p < 0.01; ***p < 0.001.

#### Effects in Academic Stress Symptoms

**FIGURE 2:**
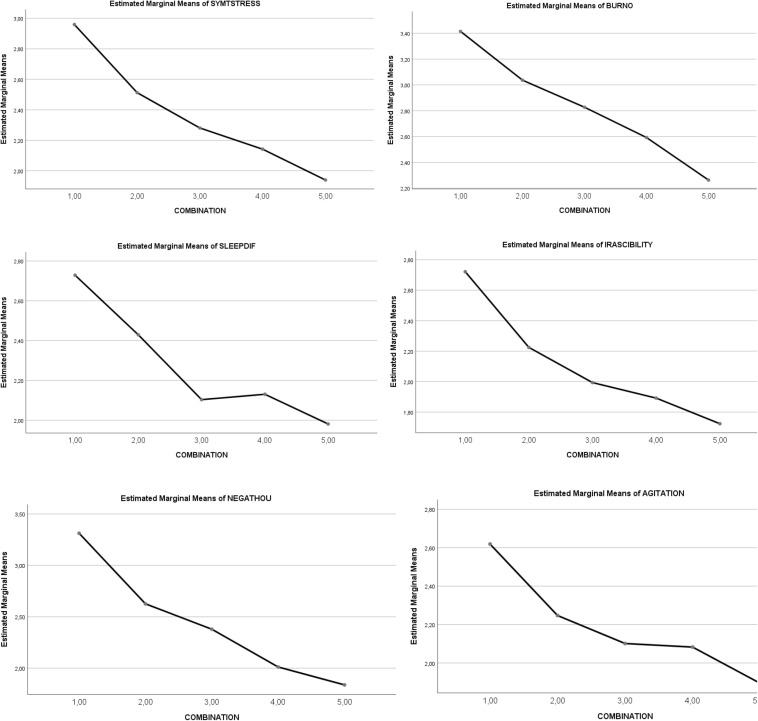
Graphical representation of the effects of the types of combinations (levels 1–5) on *academic stress symptoms:* (1) SYMTSTRESS = total stress symptoms; (2) BURNOU = burnout; (3) SLEEPDIF = sleep difficulties; (4) IRASCIBILITY = irritability; (5) NEGATHOU = negative thoughts; (6) AGITATION = agitation.

A statistically significant main effect of the *five combinations of SR and RT* was noted on the total *symptoms of academic stress* (5,4 < 3 < 2,1). The statistically significant partial effect was maintained for each factor (*burnout*, *sleep difficulty*, *irritability*, *negative thoughts*, and *agitation*). Thus, factors of academic stress symptoms progressively decrease through the five levels of combination. Overall, the clearest effect that appears is that a higher combination level leads to a decreasing level of stress symptoms. See Table 5. The graphic representation of the differential progressive effect of the combination between SR and RT levels is shown in Figure 2.

**TABLE 5:** Effects of types of combination in symptoms of stress (*n* = 401).

	1	2	3	4	5	Effects *post hoc*
		
	(*n* = 28)	(*n* = 131)	(*n* = 155)	(*n* = 109)	(*n* = 63)	
**Stress Symptoms**						
Total	2.95(0.70)	2.51(0.73)	2.28(0.65)	2.14(0.64)	1.93(0.72)	*F*(4,481) = 15.253, *p* < 0.001, η^2^ = 0.113, 5,4 < 3 < 2,1** *F*(20,1920) = 4.696, *p* < 0.001, η^2^ = 0.053
Burnout	3.41(0.72)*	3.03(0.89)	2.81(0.92)	2.59(0.90)	2.26(0.87)	*F*(4,481) = 12.649, *p* < 0.001, η^2^ = 0.095, 5,4 < 3 < 2,1**
Sleep difficulties	2.72(0.56)*	2.42(0.92)	2.10(0.80)	2.13(0.81)	1.98(0.90)	*F*(4,481) = 6.675, *p* < 0.001, η^2^ = 0.063, 5,4,3 < 2,1**
Irritability	2.72(0.99)*	2.22(0.91)	1.99(0.75)	1.99(0.75)	1.71(0.82)	*F*(4,481) = 9.735, *p* < 0.001, η^2^ = 0.075, 5 < 4,3 < 2,1**
Negative thoughts	3.31(1.0)*	2.62(1.0)	2.37(0.92)	2.01(0.77)	1.83(0.84)	*F*(4,481) = 19.068, *p* < 0.001, η^2^ = 0.137, 5,4 < 3,2 < 1**
Agitation	2.61(0.92)*	2.24(0.91)	2.10(0.80)	2.08(0.71)	1.89(0.90)	*F*(4,481) = 4.367, *p* < 0.001, η^2^ = 0.075, 5,4,3 < 2,1**

Type 1 (low personal self-regulation, and low regulatory teaching); Type 2 (low personal self-regulation and high regulatory teaching); Type 3 (medium personal self-regulation and medium regulatory teaching); Type 4 (high personal self-regulation and low regulatory teaching); Type 5 (high personal self-regulation and high regulatory teaching). *Statistical relevant effect; **p < 0.01.

## Discussion and Conclusion

The SRL vs. ERL theory (de la Fuente, 2017) predicted that the level of student self-regulation (personal) and the level of external regulation from the teaching process (context) would jointly predict stress factors and symptoms. In addition, this type of interaction could be understood as the combination of low/medium/high levels of both factors, as supported by previous evidence in this direction (de la Fuente et al., 2015b, 2017, 2019b, 2020). Complementary, the directionality of the proposed hypothesis stated that a gradual decrease in the level of regulation (internal and external) would result in a proportional increase in (1) stress factors and (2) stress symptoms. By contrast, the higher the level of internal and external regulation, the lower the level of stress factors and symptoms in undergraduate students.

The results supported the prediction of the *first hypothesis*. The evidence confirmed the differential presence of stress factors. The level of self-regulation behavior was shown to negatively predict the level of stress factors and symptoms, while the level of regulatory teaching (external regulation) also did so, though to a lesser degree.

(1) This result, showing the importance of *students’ self-regulation level* in determining the level of stress factors and symptoms, is consistent with evidence reported previously (Durand-Bush et al., 2015; Bingen et al., 2019; de la Fuente et al., 2019b). This result confirms the idea that self-regulation is a meta-behavioral variable that, due to its nature of behavior oversight, offers *protection* against academic stress and is associated with meta-affective variables like coping strategies (de la Fuente et al., 2015c) or meta-motivational variables like resilience (Artuch-Garde et al., 2017). Complementarily, this finding also shows that a lack of student self-regulation acts as a factor of *vulnerability*, predisposing to a higher level of stress factors and symptoms.
(2) As for the effect of *regulatory teaching level* on these stress factors and symptoms, results are also consistent with previous findings (de la Fuente et al., 2017, 2019b, 2020). *Regulatory teaching* is confirmed as a protective factor against factors of stress. Effective teaching decreases potential stress factors pertaining to the teaching process (inadequate methodology, lack of interesting course content, disorganization) and to the learning process (anxiety, perceived lack of control, and excessive workload), just as it predisposes to low levels of stress symptoms. By contrast, a *low level of regulatory teaching* brings with it higher levels of stress factors in the teaching process (inadequate methodology, unscheduled changes, and less meaningful content) and in the learning process (more anxiety, task overload): this is why it is considered *dysregulatory*. This is the context where greater stress symptoms appear (Khan et al., 2020). It has therefore been demonstrated, in a precise manner, that factors of the teaching process can constitute either protective or risk factors during the period of university learning (Vermunt, 2007). These results indicate a course of action for alleviating the stress factors associated with the teaching–learning context, usually present in university environments (Moffa et al., 2016; Shaw et al., 2017; Mainhard et al., 2018).

Results from testing the *second hypothesis* are very consistent with the idea that a higher combination of the two types of regulation (subject and context) significantly predicts a decrease in stress factors and symptoms. This finding is very important for the theoretical model, offering consistency with levels of negative emotionality reported in previous studies (de la Fuente et al., 2019b, 2020); empirical evidence supports the idea that the *combined levels* of individual and contextual regulation are what delimit the level of stress. Students with a lower level of self-regulation (non-regulation or dysregulation), who are also exposed to non-regulatory teaching processes (no external regulation or dysregulation), are quite consistently shown to experience the greatest factors and symptoms of stress. The opposite occurs in the case of students with high self-regulation who are exposed to teaching that is high in external regulation. These results allow us to analyze academic stress from an interactive approach, taking into consideration the combination of *stress factors* pertaining to the student and to the teaching process, whether they are factors of *protection* or of *vulnerability* to symptoms of *academic stress.* This is a step forward from analyzing these aspects independently, as has been done traditionally (Karaman et al., 2017). The five-level heuristic presented here allows for precise analysis and prediction at each level, from the most protective levels to the levels of most vulnerability.

This evidence, in addition to supporting the proposed hypotheses, constitutes progress in the conceptualization of academic stress, by taking an educational psychology approach. These results offer solid backing for contextualized, molar, psycho-educational models in real settings, taking us beyond a molecular-level understanding (de la Fuente et al., 2019a) of how personal variables affect stress factors and symptoms. This contribution should make us move toward a more precise, interactive conception of academic stress in the university. Indeed, self-regulation is a personal protective factor against academic stress in the university setting, and the lack of self-regulation is a risk factor for it. However, the lack of external regulation, likewise, is a contextual risk factor for academic stress, while external regulation is a contextual protective factor for stress. It therefore makes little sense to evaluate only one part of this binomial. If students with a low level of self-regulation perceive more stress factors inherent in the teaching process and, consequently, experience more stress symptoms, any innovation in teaching design must take into account the teaching process itself as a *protective* or *risk* factor.

### Limitations and Future Directions

Despite the evidence offered, the present study has several limitations which must be addressed in future research:

(1) Variability of the sample is limited because the participants included only undergraduate students taking subjects in degree programs that we were able to assess. Courses with other profiles are also offered at the university and should be the object of this evaluation in the future, in order for conclusions to be generalized. Sample size and variability can be increased by including university students majoring in different fields of study. There is also a sample limitation referring to the large majority of female participants.
(2) A methodological limitation of this study is its reliance on collecting student data by means of self-report systems. Collecting data that are based only on student perceptions can lead to limitations and biases. In the future, other assessments could be incorporated, including the perspective of the teacher giving the course, in order to compare student and teacher perceptions of the process under assessment. The instrument used here offers assessment options for both teachers and students to evaluate the same teaching–learning processes (de la Fuente et al., 2012).
(3) Finally, another aspect to be considered is the research design. Although the existing design addressed the stated objectives and is ecologically valid, in the future, other types of complementary designs for analyzing this area need to be considered, thereby obtaining other important information. A multi-method, multi-technique evaluation system always improves research on psychological problems.

### Practical Implications for Educational Psychology

When implementing improvements in the university teaching process, we should consider what kind of context is being designed, within the framework of SRL vs. ERL theory (de la Fuente, 2017). The concept of regulatory teaching is characteristic of high levels of effective teaching (protective factor against stress), while non-regulatory or dysregulatory teaching would be typical of ineffective teaching (risk factor for stress). When the teaching context does not help regulate the student’s learning, or is even dysregulatory, the student’s learning process is inadequately supported, especially if students have low self-regulation. In the absence of external regulation, students must exercise even more effort to self-regulate, in order to compensate for the lack of external help. Some previous evidence has reported results consistent with this idea (Bingen et al., 2019). This view of *academic stress* is quite novel compared to the typical view where classical stress models (Folkman and Lazarus, 1984) are merely applied in a linear way to the university context. Such a linear application attempts to explain stress at university without entering into the academic processes of teaching and learning. This is nothing other than a decontextualization of the problem of academic stress. The educational psychology point of view, offered in this study, seeks to overcome this limitation.

## Data Availability Statement

The datasets generated for this study are available on request to the corresponding author.

## Ethics Statement

The studies involving human participants were reviewed and approved by Comite de Ética University of Navarra (Spain). The patients/participants provided their written informed consent to participate in this study.

## Author Contributions

JF: conceptualization, design, and data analysis. FP-S and JM-V: foundation and initial writing. PS and LZ: English revision and final writing. AG-U: revision of the general structure of the manuscript. All authors contributed to the article and approved the submitted version.

## Conflict of Interest

The authors declare that the research was conducted in the absence of any commercial or financial relationships that could be construed as a potential conflict of interest.

## Footnotes

1 de la Fuente, J., Sander, P., Kauffman, D., and Yilmaz-Soylu, M. (2020). Differential effects of self- vs. external- regulation on learning approaches, academic achievement and satisfaction in undergraduate students. Front. Psychol. 11 (in review).

2 http://www.estres.investigacion-psicopedagogica.org/espanol/seccion.php?idseccion=10
